# Refractive Error among Male Primary School Students in Jazan, Saudi Arabia: Prevalence and Associated Factors

**DOI:** 10.2174/1874364101812010264

**Published:** 2018-09-28

**Authors:** Tariq Al Bahhawi, Anwar M Makeen, Hadi Hassan Daghreeri, Mohannad Faisal Tobaigy, Abdulrahman Mohammed Adawi, Faisal Mohammed Guhal, Murad Abdullah Akkur, Mohsen Jaber Alotayfi, Mutaz Mohammed Otayf, Meshal Salem Bajoned, Mohamed Salih Mahfouz

**Affiliations:** 1 Department of Community Medicine, Faculty of Medicine, Jazan University, Jazan, Kingdom of Saudi Arabia; 2 Faculty of Medicine, Jazan University, Jazan, Kingdom of Saudi Arabia

**Keywords:** Refractive Error, Myopia, Hyperopia, Jazan region, Prevalence and associated factors, Optometrist

## Abstract

**Background:**

Refractive error is a common and serious eye disorder that affects more than 153 million people globally. The aim of this study was to estimate the prevalence and pattern of refractive error among male primary school children in Jazan region, Saudi Arabia.

**Methods:**

A cross-sectional study was conducted among a randomly selected group of 395 students (aged 6-14 years) in Jazan region, Southwest Saudi Arabia. An optometrist and medical students assessed the refraction error using an autorefractor, a Snellen E chart and retinoscopy.

**Results:**

The overall prevalence of uncorrected refractive error in either eye was, 22% higher among rural students. The most prevalent refractive error was hyperopia (32.2%) followed by myopic astigmatism (31%) then myopia (17.2%). Next were hyperopic astigmatism (16.1%) and mixed astigmatism (3.5%). The following variables were associated with a higher risk of refractive errors and myopia: living in rural areas, having parents with refractive errors, spending more time on electronic devices and shorter visual distances.

**Conclusion:**

Refractive error was highly prevalent among primary school children in Jazan, Saudi Arabia. The rural students were more affected by refractive errors, mainly hyperopia. The preschool vision test should be reconsidered, and a periodic vision examination should be applied to detect vision problems as early as possible.

## 1 INTRODUCTION

The WHO has estimated that about 153 million people over the age of five years are visually impaired as a result of uncorrected Refractive Error (RE) [1]. Around 12.8 million children (aged 5 to 15 years) are visually impaired, with a global prevalence of 1%, due to uncorrected or inadequately corrected refractive errors [1].

The WHO and 20 international Non-Governmental Organizations (NGOs) launched the global initiative, “Vision 2020,” in 1999 with the aim of eliminating avoidable blindness by the year 2020 [2].

Visual impairment due to uncorrected refractive errors can lead to short-term and long-term consequences in adults and children. Examples include lost educational and career opportunities lost economic benefits for individuals, families and societies, and poor quality of life [1, 3]. Moreover, the uncorrected refractive error could decrease a child’s interaction and learning in the classroom, negatively affecting his or her learning process [4].

In Saudi Arabia, many studies have been conducted to estimate the prevalence and pattern of refractive error among primary school children. A study conducted in Abha city revealed an RE prevalence of 23%, while one conducted on pre-school children in Jeddah city showed an RE prevalence of 10.7% [5, 6]. Desouky and Tariq-Khan (2014) conducted a study in Taif City that showed a 16.4% RE prevalence in a sample of female primary school children [7]. Moreover, Wadaani *et al*. (2013) conducted a study on primary school children in Al Hassa region that revealed a RE prevalence of 13.7% [8].

Although many studies have been conducted on refractive error in Saudi school children, a literature search suggested that no previous study had been conducted to measure the prevalence of refractive errors among children in Jazan region. Hence, the aim of the present study was to estimate the prevalence and pattern of refractive error among primary school children in Jazan region, Saudi Arabia.

## 2 METHODS

### 2.1 Design and Setting

A cross-sectional study was conducted among male primary school students (age range: 6-14 years) in Jazan region in October 2015. Jazan region is one of the smallest regions in Saudi Arabia; it is located in Southwest Saudi Arabia and stretches along the Red Sea coast for 300 km, immediately north of Yemen. The total population of the region was about 1,500,000 in the year 2015 [9].

### 2.2 Sampling Procedure

The sample size for this study was determined to be 500 students based on a sample size formula for a cross-sectional study design [10]. The following parameters were used for sample size estimation: *p* = 50%, 95% Confidence Interval (C.I.), an error below 5% and a non-response rate of 10%. The proposed sampling design was a multistage sampling method that divided Jazan region into two educational sections-the Jazan and Sabya general educational departments. The Jazan general educational department was randomly selected. Then it was divided into six educational centers based on Ministry of Education segmentation. Two primary schools from each educational sector were randomly selected then lists of children at each school were used as sample frames for the simple random sampling method.

### 2.3 Demographics and Vision Data

For other demographic and risk factor information, a short questionnaire was designed, and parents or guardians responded to it. The questionnaire sought information on details such as the child’s personal data, father’s and mother’s levels of education, family income, internet use, watching television (TV), playing computer games and family history of RE.

### 2.4 Refractive Error Examinations

The examinations conducted involved three main steps: first was the examination of visual acuity using a Snellen C chart. Next was the autorefractor test (ACCUREF R-800) to measure the spherical equivalent and the cylindrical equivalent. Last was the non-cycloplegic retinoscopic examination by a trained optometrist.

### 2.5 Snellen Chart and Autorefractometer

All the children underwent full uncorrected visual acuity assessment. Each child was positioned 6 meters away from a well-lit C chart. The child's right eye was examined while the left eye was covered, and vice versa. Then the child was instructed to identify the optotype on the monitor. The smallest line on which the child could read more than half of the letters was recorded. The autorefractor examination was done by placing each child’s chin on the chin holder and asking him to view the image with both eyes.

### 2.6 Non-Cycloplegic Retinoscopy

The optometrist used a streak retinoscope to confirm the results. The children’s spherical equivalent (SE) was considered as the mean and the SE calculated using the standard formula (SE = sphere + (cylinder/2)). The definitions of refractive error were based on the following categories: myopia ≤- 0.75 diopters (D), low myopia ≤- 0.75 D to ≥- 3 D, moderate myopia ≤-3 D to ≥-6 D, high myopia ≤- 6 D, hyperopia ≥ 1 D, high hyperopia ≥ 3 D and astigmatism ≥ 1 D [11].

### 2.7 Data Analysis

Data was entered and analyzed using SPSS version 20 (SPSS Inc., Chicago, IL, USA). Data analysis involved descriptive statistics as well as some techniques of inferential statistics. The descriptive statistics included a simple tabulation, frequencies and cross-tabulations. The chi-squared test was used to test differences in proportion and Odds Ratios (ORs), and their 95% Confidence Intervals (CIs) were used as indicators of strength of association. A *p*-value of less than 0.05 was used as the cut-off level for statistical significance.

## 3 RESULTS

The informed consent form and the study questionnaire were distributed to 500 students in all the selected governorates. A total of 395 (79%) requests for consent received approval: the parents of the students signed them. The demographic characteristics of the students are presented in Table **1**. According to the table, the students’ ages ranged from 6-14 years, and rural students represented 70.1% of the total number of students.

The total number of students with refractive errors was *n*=87, with a prevalence of 22% (95% CI 18.2-26.4). The percentage of affected students in rural areas was 24.6% (95% CI 19.9-30.1) compared to 15.5% (95% CI 10.1-23.2) in urban areas; a significant difference existed between them (*p*-value = 0.047). The rest of the data in the table did not show significant differences where the different characteristics were concerned as the *p-*values were higher than 0.05. We found that refractive errors affected both eyes at a percentage of 89%, while they affected the right eye alone at a rate of 7% and the left eye alone at a rate of 4% (Table **2**).

Table **3** presents the prevalence of myopia, hyperopia and myopic and hyperopic astigmatism with demographic characteristics. Among students with refractive error, hyperopia was the most common refractive error in the sample (32.2%) followed by myopic astigmatism (31%) then myopia (17.2%). Hyperopic astigmatism (16.1%) and mixed astigmatism (3.4%) followed.

Table **4** illustrates the association between refractive errors and certain risk factors. A positive association with the presence of refractive errors was observed among children who spent more hours watching TV and among students who used electronic devices. Regarding family history of refractive errors, the results showed a significant positive association between children's refractive errors and the status of parents' vision problems with an odds ratio of 1.71. There was an association between students watching TV from a close distance and refractive error with an odds ratio of less than one for large distances from the TV as large distances decreased the odds of refractive error. The students in the rural areas were significantly more than twice as likely to be affected by refractive errors as the students in the urban areas. The table also indicated that the same factors associated with the refractive errors showed similar relationships with myopia.

## 4 DISCUSSION

Refractive error is a major challenge facing primary school children in Saudi Arabia [5-8] and globally [12-15] as many children may not know that they have visual problems and may think that they have normal sight. Some students may have trouble reading the board or seeing close objects clearly, which might affect their academic performance and their quality of life.

To the best of our knowledge, this is the first study to address the issue of prevalence and factors associated with Refractive Error (RE) among school children in the Jazan region. The present study revealed the overall prevalence of refractive errors in either eye of 22%, which was very close to 23%, the prevalence that the study conducted in Abha city arrived at [5]. This prevalence is higher than those that other studies conducted in Saudi Arabia (in Al Hassa, Jeddah and Taif City) arrived at [8, 6, 7].

Upon the comparison of our estimates with those of other studies, we found them to be similar to those of a study conducted in Egypt (22.1%) [14] and higher than those of studies conducted in countries like Qatar (19.7%) [16], Nepal (8.6%) [17] and India (13.09%) [18]. Our prevalence was also lower than Taiwan’s and Srinagar’s [19, 20]. The variation in prevalence may have resulted because we conducted this study on a male population only. By contrast, other studies were conducted on both sexes.

Despite the fact that myopia is the most prevalent refractive error globally, our results showed that hyperopia was the most prevalent refractive error in our sample [1]. Moreover, most studies conducted in Saudi Arabia showed that myopia was the most prevalent refractive error [8, 21]. A recent study conducted in Jazan supported our findings, which revealed that hyperopia was more prevalent than myopia [22].

Our results showed that hyperopia was the most common refractive error (32.2%) of all, followed by myopic astigmatism (31%), myopia (17.2%), hyperopic astigmatism (16.1%) and mixed astigmatism (3.5%). On the other hand, the study that Waddani *et al*. (2013) conducted in Al Hassa [8] found the most prevalent refractive error to be myopia (65.6%) followed by myopic astigmatism (12.4%), hyperopic astigmatism (12%) and hyperopia (9.8%). The differences could be attributed to the differentials in socioeconomic conditions, variations in the operational definitions, cut-off points of refractive errors and factors related to environmental influences.

In contrast to many studies [23-25], our results showed that refractive errors affected the rural population more than the urban population. Regarding the associations between refractive errors and selected risk factors, the data of our study support the findings of other studies. We found an association between refractive errors and parental history of refractive errors that was in line with the findings of Ip *et al*. (2007) and Zadnik *et al*. (1994) [26, 27]. We also found an association between time spent on electronic devices and refractive errors, and this was well documented by [28, 29]. Moreover, shorter distances from the TV screen were emphasized by [26, 29], and our results revealed an association between short distances from the TV screen and the increased risk of refractive errors and myopia.

The lack of cycloplegic objective refraction was a major limitation of this study. We should be very careful when interpreting the results of this study, for cycloplegia is truly the gold standard for the diagnosis of refractive errors. It is regrettable that such a procedure was not performed. The reason for this failure was that the procedure would have blurred the students’ vision for 3-8 hours. The intrinsic value of this study would have increased immensely had cycloplegic drops been used for a reliable objective refraction. Secondly, we conducted our study on male students alone for some practical reasons. Finally, the study was based on a cross-sectional study design, so the risk factors should be interpreted carefully.

## CONCLUSION

Refractive error was highly prevalent among primary school children in Jazan, Saudi Arabia. The rural students were more affected by refractive errors, mainly hyperopia. We recommend a study with cycloplegic to assess the issue of RE in greater depth. The preschool vision test should be reconsidered, and a periodic vision examination should be applied to detect vision problems as early as possible.

## Figures and Tables

**Table 1:** Demographic characteristics of the study population (n=395).

**Characteristic**	**N**	**%**
**Place of residence (n= 388)**	–	–
Rural	272	70.1
Urban	116	29.9
**Age group (years)** **(n = 385)**	–	–
6-8	130	33.8
9-11	204	53
12-14	51	13.2
**Mother’s level of education (n=390)**	–	–
Illiterate	68	17.4
Basic education	200	51.3
University and above	122	31.3
**Father’s level of education (n=392)**	–	–
Illiterate	31	7.9
Basic education	227	57.8
University and above	134	34.2
**Monthly income (SAR) (n=372)**	–	–
Less than 5,000	115	30.9
5,000-10,000	106	28.5
More than 10,000	151	40.6
**Classes (n=389)**	–	–
First	56	14.4
Second	56	14.4
Third	74	19
Fourth	78	20.1
Fifth	63	16.2
Sixth	62	15.9

**Table 2:** Prevalence of refractive error with regard to some selected characteristics.

**Characteristic**	**Students with RE**		*p*-value*
	**N (%)**	**95% CI**	
**Overall Prevalence**	87(22.0)	(18.2-26.4)	–
**Place of residence**	–	–	0.047
Rural	67(24.6)	(19.9-30.1)	
Urban	18(15.5)	(10.1-23.2)	
**Age group (years)**	–	–	0.640
6-8	31(23.8)	(17.4-31.9)	
9-11	43(21.1)	(16.1 -27.2)	
12-14	9(17.6)	(9.6-30.3)	
**Mother’s education status**	–	–	0.168
Illiterate	7(10.3)	(5.1-19.8)	
Basic education	46(23)	(17.7-29.3)	
University and above	31(25.4)	(18.5-33.8)	
**Father’s education status**	–	–	0.316
Illiterate	6(19.4)	(9.3-36.4)	
Basic education	45(19.8)	(15.2-25.5)	
University and above	34(25.4)	(18.8-33.4)	
**Monthly income (SAR)**	–	–	0.389
Less than 5,000	20(17.4)	(11.6-25.4)	
5,000-10,000	28(26.4)	(19-35.6)	
More than 10,000	36(23.8)	(17.8-31.3)	
**Grades**	–	–	0.737
First	15(26.8)	(17-39.7)	
Second	12(21.4)	(12.7-33.9)	
Third	12(16.2)	(9.6-26.3)	
Fourth	16(20.5)	(13.1-30.8)	
Fifth	16(25.4)	(16.3-37.4)	
Sixth	13(21)	(12.7-32.7)	

• *Based on chi-squared test*

**Table 3:** Prevalence of myopia, hyperopia and myopic and hyperopic astigmatism with demographic variables.

**Variables**	**Myopia NO (%)**	**Hyperopia NO (%)**	**Myopic AST** **NO (%)**	**Hyper. AST NO (%)**	**MIX. AST NO (%)**	***p*-value**
**Age Group (years) (n=83)**	–	–	–	–	–	0.963
6-8	4(12.9)	10(23.3)	9(29.0)	6(19.4)	2(6.2)	
9-11	9(20.9)	14(32.6)	13(30.2)	6(14.0)	1(2.3)	
12-14	2(22.2)	3(33.3)	3(33.3)	1(11.1)	0(0.0)	
**Place of Residence (n=86)**	–	–	–	–	–	0.082
Rural	15(22.4)	21(31.3)	20(29.9)	10(14.9)	1(1.5)	
Urban	0(0.0)	6(31.6)	7(36.8)	4(21.1)	2(10.5)	
**Classes (n=83)**	–	–	–	–	–	0.485
First	2(13.3)	5(33.3)	5(33.3)	1(6.7)	2(13.3)	
Second	1(8.3)	5(41.7)	4(33.3)	2(16.7)	0(0.0)	
Third	0(0.0)	4(33.3)	4(33.3)	4(33.3)	0(0.0)	
Fourth	6(37.5)	6(37.5)	3(18.8)	1(6.3)	0(0.0)	
Fifth	2(12.5)	5(31.3)	6(37.5)	2(12.5)	1(6.3)	
Sixth	3(25.0)	3(25.0)	4(33.3)	2(16.7)	0(0.0)	
**Mother’s Education Status (n=84)**	–	–	–	–	–	0.295
Illiterate	1(14.3)	3(42.9)	2(28.6)	0(0.0)	1(14.3)	
Basic Education	6(13.0)	16(34.8)	14(30.4)	9(19.6)	1(2.2)	
University & above	8(25.8)	9(29.0)	11(35.5)	3(9.7)	0(0.00	
**Father’s Education Status (n=85)**	–	–	–	–	–	0.285
Illiterate	1(16.7)	2(33.3)	1(16.7)	1(16.7)	1(16.7)	
Basic Education	6(13.3)	19(42.2)	11(24.4)	8(17.8)	1(2.2)	
University & above	8(23.5)	7(20.6)	14(41.2)	4(11.8)	1(2.9)	
**Monthly Income (SAR) (n=85)**	–	–	–	–	–	0.191
Less than 5,000	3(14.3)	5(23.8)	11(52.4)	1(4.8)	1(4.8)	
5,000-10,000	4(14.3)	12(42.9)	5(17.9)	7(25.0)	0(0.0)	
>10,000	8(22.2)	11(30.6)	10(27.8)	6(16.7)	1(2.8)	
**Overall Prevalence**	**15(17.2)**	**28(32.2)**	**27(31.0)**	**14(16.1)**	**3(3.4)**	

**Table 4:** Association between refractive errors and certain risk factors.

**Risk Factor**	**Refractive Errors**			**Myopia**		
	**OR**	**(95% CI)**		**OR**	**(95% CI)**	
		**Lower**	**Upper**		**Lower**	**Upper**
**Age Groups (years)**	–	–	–	–	–	–
6-8	1.00	–	–	1.00	–	–
9-11	1.12	0.69	1.81	0.93	0.50	1.75
12-14	1.44	0.69	3.05	1.21	0.45	3.23
**Mode of Living**	–	–	–	–	–	–
Urban	1.00	–	–	1.00	–	–
Rural	2.70	1.55	4.68	3.93	1.63	9.46
**Use of Electronic Devices (ED)**	–	–	–	–	–	–
Non-users	1.00	–	–	1.00	–	–
Users	1.40	0.75	2.61	2.71	0.94	7.78
**Hours Using Elect. Devices**	–	–	–	–	–	–
Less than 2 Hours	1.00	–	–	1.00	–	–
3-5 Hours	2.66	0.90	7.86	3.46	1.10	10.90
More than 5 Hours	1.88	0.56	6.29	3.10	0.80	12.03
**Outdoor Activities**	–	–	–	–	–	–
Yes	1.00	–	–	–	–	–
NO	1.42	0.75	2.69	1.88	0.65	5.46
**Family History of RE**	–	–	–	–	–	–
No	1.00	–	–	1.00	–	–
Yes	1.71	1.09	2.67	2.92	1.55	5.48
**Distance to TV Screen**	–	–	–	–	–	–
Close	1.00	–	–	1.00	–	–
Average	0.56	0.24	1.28	0.59	0.21	1.68
Far	0.48	0.22	1.04	0.72	0.27	1.96

## References

[1] Pascolini D., Mariotti S.P. (2012). Global estimates of visual impairment: 2010.. Br. J. Ophthalmol..

[2] Pizzarello L., Abiose A., Ffytche T., Duerksen R., Thulasiraj R., Taylor H., Faal H., Rao G., Kocur I., Resnikoff S. (2004). VISION 2020: The right to sight: A global initiative to eliminate avoidable blindness.. Arch. Ophthalmol..

[3] Fricke T.R., Holden B.A., Wilson D.A., Schlenther G., Naidoo K.S., Resnikoff S., Frick K.D. (2012). Global cost of correcting vision impairment from uncorrected refractive error.. Bull. World Health Organ..

[4] Negrel A.D., Maul E., Pokharel G.P., Zhao J., Ellwein L.B. (2000). Refractive error study in children: Sampling and measurement methods for a multi-country survey.. Am. J. Ophthalmol..

[5] Abolfotouh M., Faheem Y., Badawi I., Khairallah S. (1993). Prevalence of refractive errors and their optical correction among schoolboys in Abha City, Asir Region, Saudi Arabia.. Health Ser. J. Eastern Mediterran..

[6] Wedad M. (2002). Bardisi, Bakr M., bin Sadiq, Vision screening of preschool children in Jeddah, Saudi Arabia.. Saudi Med. J..

[7] Desouky D.E, Nighat M, Tariq K. (2014). Refractive error among a sample of female primary school children in Taif City, KSA.. Inter J of Public Health and Epidemiol.

[8] Al Wadaani F.A., Amin T.T., Ali A., Khan A.R. (2012). Prevalence and pattern of refractive errors among primary school children in Al Hassa, Saudi Arabia.. Glob. J. Health Sci..

[9] http://www.stats.gov.sa/en/4250.

[10] Lwanga S.K., Lemeshow S. (1991). Sample size determination in health studies: A practical manual http://apps.who. int/iris/handle/10665/40062.

[11] Williams K.M., Verhoeven V.J.M., Cumberland P., Bertelsen G., Wolfram C., Buitendijk G.H., Hofman A., van Duijn C.M., Vingerling J.R., Kuijpers R.W., Höhn R., Mirshahi A., Khawaja A.P., Luben R.N., Erke M.G., von Hanno T., Mahroo O., Hogg R., Gieger C., Cougnard-Grégoire A., Anastasopoulos E., Bron A., Dartigues J.F., Korobelnik J.F., Creuzot-Garcher C., Topouzis F., Delcourt C., Rahi J., Meitinger T., Fletcher A., Foster P.J., Pfeiffer N., Klaver C.C., Hammond C.J. (2015). Prevalence of refractive error in Europe: The European Eye Epidemiology (E(^3^)) Consortium.. Eur. J. Epidemiol..

[12] Sewunet S.A., Aredo K.K., Gedefew M. (2014). Uncorrected refractive error and associated factors among primary school children in Debre Markos District, Northwest Ethiopia.. BMC Ophthalmol..

[13] Rahman M., Devi B., Kuli J.J., Gogoi G. (2015). A study on the refractive status of school going children aged between 10 to 15 years in Dibrugarh Town, Assam, India.. IOSR J Dent Med Sci.

[14] EL-Bayoumy BM (2007). Saad A, Choudhury AH. Prevalence of refractive error and low vision among school children in Cairo.. East. Mediterr. Health J..

[15] Pavithra M.B., Maheshwaran R., Rani Sujatha M.A. (2013). A study on the prevalence of refractive errors among school children of 7–15 years age group in the field practice areas of a medical college in Bangalore.. Int. J. Med. Sci. Public Health.

[16] Bener A., Al-Mahdi H.S. (2012). Internet use and television viewing in children and its association with vision loss: A major public health problem.. J. Public Health Africa.

[17] Pokharel A., Pokharel P.K., Das H., Adhikari S. (2010). The patterns of refractive errors among the school children of rural and urban settings in Nepal.. Nepal. J. Ophthalmol..

[18] Singh H., Saini V.K., Yadav A., Soni B. (2013). Refractive errors in school going children: Data from a school screening survey programme.. Natl. J. Community Med..

[19] Lin L.L., Chen C.J., Hung P.T., Ko L.S. (1988). Nation-wide survey of myopia among schoolchildren in Taiwan, 1986.. Acta Ophthalmol. Suppl..

[20] Khan A.A., Nasti A.R., Dar M.A., Lone S.A. (2005). Prevalence of refractive errors in school children.. JK Pract..

[21] Aldebasi YH (2014). Prevalence of correctable visual impairment in primary school children in Qassim Province, Saudi Arabia.. J Optometry.

[22] Darraj A, Barakat W, Kenani M, Shajry R, Khawaji A, Bakri S, Makin A, Mohanna A, Yassin AO (2016). Common eye diseases in children in Saudi Arabia (Jazan).. Ophthalmology and eye diseases.

[23] He M., Zeng J., Liu Y., Xu J., Pokharel G.P., Ellwein L.B. (2004). Refractive error and visual impairment in urban children in southern china.. Invest. Ophthalmol. Vis. Sci..

[24] Zhao J., Pan X., Sui R., Munoz S.R., Sperduto R.D., Ellwein L.B. (2000). Refractive error study in children: Results from Shunyi district, China.. Am. J. Ophthalmol..

[25] Dandona R., Dandona L. (2003). Childhood blindness in India: A population based perspective.. Br. J. Ophthalmol..

[26] Ip J.M., Huynh S.C., Robaei D., Rose K.A., Morgan I.G., Smith W., Kifley A., Mitchell P. (2007). Ethnic differences in the impact of parental myopia: Findings from a population-based study of 12-year-old Australian children.. Invest. Ophthalmol. Vis. Sci..

[27] Zadnik K., Satariano W.A., Mutti D.O., Sholtz R.I., Adams A.J. (1994). The effect of parental history of myopia on children’s eye size.. JAMA.

[28] Mutti DO, Mitchell GL, Moeschberger ML, Jones LA, Zadnik K Parental myopia, near work, school achievement, and children's refractive error..

[29] Hsu C.C., Huang N., Lin P.Y., Tsai D.C., Tsai C.Y., Woung L.C., Liu C.J. (2016). Prevalence and risk factors for myopia in second-grade primary school children in Taipei: A population-based study.. J. Chin. Med. Assoc..

